# Supplementary material for: Community detection in brain connectomes with hybrid quantum computing
Source: Sci Rep. 2023 Mar 1;13:3446. doi: 10.1038/s41598-023-30579-y (PMC9977923; doi:10.1038/s41598-023-30579-y)
Supplement: Supplementary file 1 — Supplementary Information 1. [file 41598_2023_30579_MOESM1_ESM.pdf]

Supplementary Material

Network	Nodes	Edges	Average Degree	Clustering Coefficient	Average Degree Centrality
Karate club	34	78	4.58	0.57	0.14
AAL90	90	441	9.14	0.55	0.10
Dosenbach	160	891	11.13	0.36	0.07

Table S1. Summary of the properties of the used networks

Network	Relative Increase [MEAN $\pm$ SEM] (%)	Cohen's d
Karate club	0.90 $\pm$ 1.6 (%) (p=0.284)	0.812
AAL90	0.53 $\pm$ 0.46 (%) (p=0.125)	1.639
Dosenbach	3.04 $\pm$ 1.53 (%) (p=0.021)	2.913

Table S2. Exact results for Fig. 6. Statistical significance is assessed by means of one-tailed Welch's t-test, as we hypothesize a positive effect in the modularity gain when using Quantum Annealing. Cohen's d is a measure of the effect size present in the two populations without assessing the statistical significance.

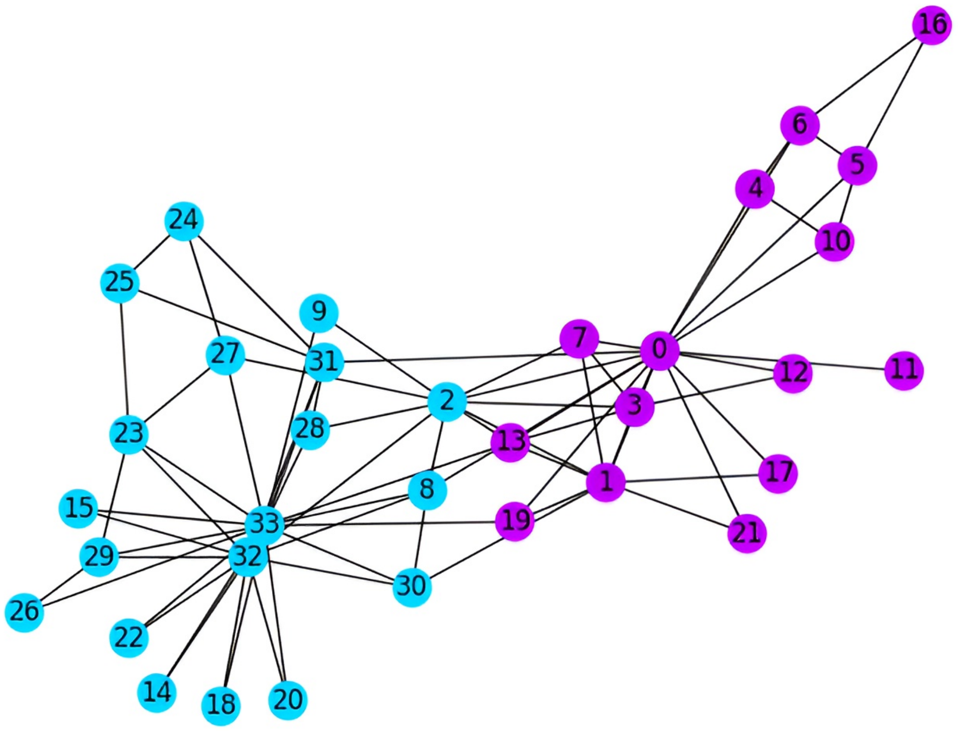

Figure S1. Ground-truth labeling for two communities in the Karate club[35]

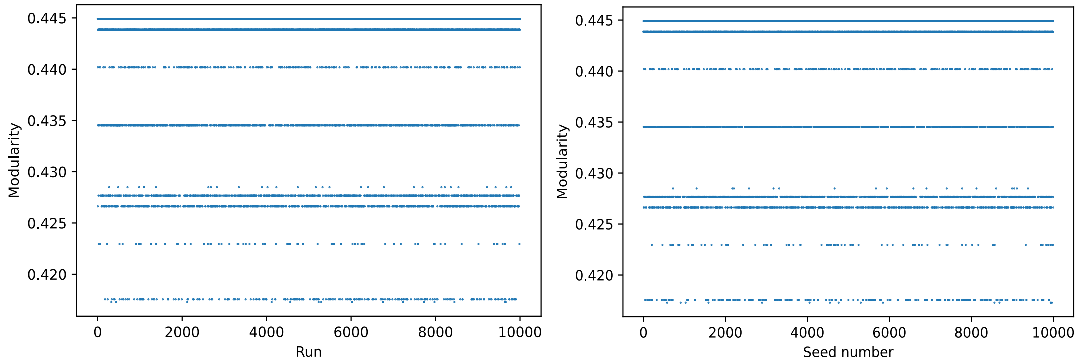

Figure S2. Left: Modularities after LCDA in NetworkX results for 10000 different runs with seed number equals to 123. Right: Modularities after LCDA in NetworkX for 10000 different seeds. Ech point represents the average of 100 different runs with the same seed (i.e., same initialization).
